# Antioxidative Activities of Micronized Solid-State Cultivated *Hericium erinaceus* Rich in Erinacine A against MPTP-Induced Damages

**DOI:** 10.3390/molecules28083386

**Published:** 2023-04-12

**Authors:** Chun-Hsien Hsu, En-Chih Liao, Win-Chin Chiang, Kai-Lee Wang

**Affiliations:** 1 Department of Family Medicine, Taipei City Hospital, Heping Fuyou Branch, Taipei 100, Taiwan; 2 Department of Family Medicine, Cardinal Tien Hospital, New Taipei 231, Taiwan; 3 School of Medicine, College of Medicine, Fu Jen Catholic University, New Taipei 242, Taiwan; 4 General Education Center, University of Taipei, Taipei 100, Taiwan; 5 Department of Medicine, MacKay Medical College, New Taipei 252, Taiwan; 6 Institute of Biomedical Sciences, MacKay Medical College, New Taipei 252, Taiwan; 7 Jowin Biopharma Inc., New Taipei 221, Taiwan; 8 Department of Nursing, Ching Kuo Institute of Management and Health, Keelung 203, Taiwan

**Keywords:** hericium erinaceus mycelium, erinacine A, antioxidant, Parkinson’s disease, reactive oxygen species

## Abstract

The Lion’s mane mushroom (Hericium erinaceus, HE) is a traditional medical mushroom with high nutritional and economic value. HE possesses anticancer, antimicrobial, antioxidant, immunomodulating, neurotrophic, and neuroprotective activities. The present study evaluated the protection and antioxidative activities of micronized mycelium of HE (HEM) in mice treated with 1-methyl-4-phenylpyridinium (MPTP). HEM was cultivated via solid-state fermentation and micronized using cell wall-breaking technology to increase its bioavailability when ingested. Erinacine A, the bioactive compound in the HEM, played a pivotal role in antioxidant defense. We found that micronized HEM could recover the dopamine level in the mice striatum in a dose-dependent manner that had been greatly reduced during MPTP treatment. Moreover, the malondialdehyde (MDA) and carbonyl levels were reduced in the livers and brains of the MPTP + HEM-treated groups compared with the MPTP group. Additionally, antioxidant enzyme activities, including catalase, superoxide dismutase (SOD), glucose-6-phosphate dehydrogenase (G6PDH), and glutathione reductase (GRd), were elevated after the administration of HEM in MPTP-treated mice in a dose-dependent manner. Taken together, our data indicate that HEM cultivated via solid-state fermentation and processed with cell wall-breaking technology showed an excellent antioxidant efficacy.

## 1. Introduction

Neurological and neurodegenerative diseases, such as Parkinson’s disease (PD), Alzheimer’s disease (AD), and Huntington’s disease, are highly debilitating and pose significant threats to public health [1]. Considering the increasing older population worldwide, neurodegenerative diseases are bound to increase over time, especially since no medication has become available to prevent or reverse the neurodegeneration induced by these diseases. Various studies have underlined the role of oxidative stress and mitochondrial impairment on initiating the cascade of events leading to degeneration of dopaminergic neurons [2]. PD is characterized by the progressive loss of dopaminergic neurons, at least partly due to increased reactive oxygen species (ROS) in mitochondria, lipids peroxidation, DNA abnormalities, and proteins oxidation [3,4]. Toxicants that can increase oxidative stress of the substantia nigra, such as 1-methyl-4-phenyl-1,2,3,6-tetrahydropyridine (MPTP), have been used to induce PD in mice. Therefore, antioxidants capable of counteracting oxidative stress may provide a novel potential therapy to combat PD [5].

*Hericium erinaceus* (HE), also known as Lion’s mane mushroom or monkey’s head mushroom, is a widespread pharmaceutical and edible fungus found in several Asian countries. The use of HE is safe and harmless even for extended periods of time, and is traditionally used to treat peptic ulcers and acute gastritis [6]. HE contains a large number of bioactive compounds, including alkaloids, flavonoids, terpenes, polysaccharides, and metal-chelating agents [7]. Recent studies have demonstrated that HE and its extracts possess a wide range of benefits, such as anticancer, antimicrobial, antidiabetic, antioxidant, antiaging, antihyperglycemic, antihyperlipidemic, gastroprotective, immunomodulating, and neuroprotective activity [8,9,10,11].

Pertaining to its neuroprotective effects, which have been associated with the JNK/p38/NF-κB/CHOP/Fas/Bax signaling pathways [12], HE has been suggested to interrupt the apoptosis cascade by inhibiting ROS production [13,14]. HE has also been found to reduce anxiety and depression through the promotion of hippocampal neurogenesis [15]. HE and its bioactive ingredients can promote nerve growth factor expression, thereby improving cognitive impairments such as PD and AD [16]. A recent study has also confirmed that PD-induced neuroinflammation and oxidative stress could be inhibited by HE [17]. Erinacine A (EA), a bioactive compound extracted by ethanol from HE, passes through the blood–brain barrier and possesses neuroprotective properties by ameliorating lipopolysaccharide-induced inflammation [18,19]. EA also provided protection from neurotoxicity by alternating the apoptosis and cell death signaling pathways [20]. It has also been confirmed that EA stimulates the production of the nerve growth factor from astroglia, thereby promoting and maintaining neural growth [21]. These studies clearly demonstrate that HE possesses distinct neuroprotective activity.

In the present study, HE mycelia (HEM), which is cultivated under solid-state fermentation, was micronized to increase its bioavailability. The protective and antioxidant activities of HEM were evaluated in male C57BL/6Narl mice under MPTP treatment.

## 2. Results

### 2.1. MPTP Animal Model Set Up

**Figure 1:**
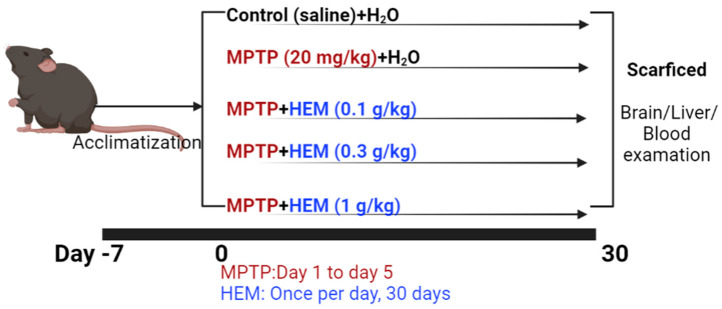
Treatment flow chart.

The MPTP model of PD was induced, as described previously [22]. Mice were randomly assigned into five groups, as shown in Figure 1: the control group, the MPTP group (20 mg/kg/day for the first 5 days; Tokyo Chemical Industry, TCI, Tokyo, Japan), and MPTP + different dosages of HEM groups (0.1 g/kg, 0.3 g/kg, and 1 g/kg, respectively). Mice received intraperitoneal (i.p.) injection of MPTP, and the same quantity of saline was given in the control group. Mice were orally gavaged with H_2_O or HEM for 30 days.

### 2.2. Particle Size Analysis

**Figure 2:**
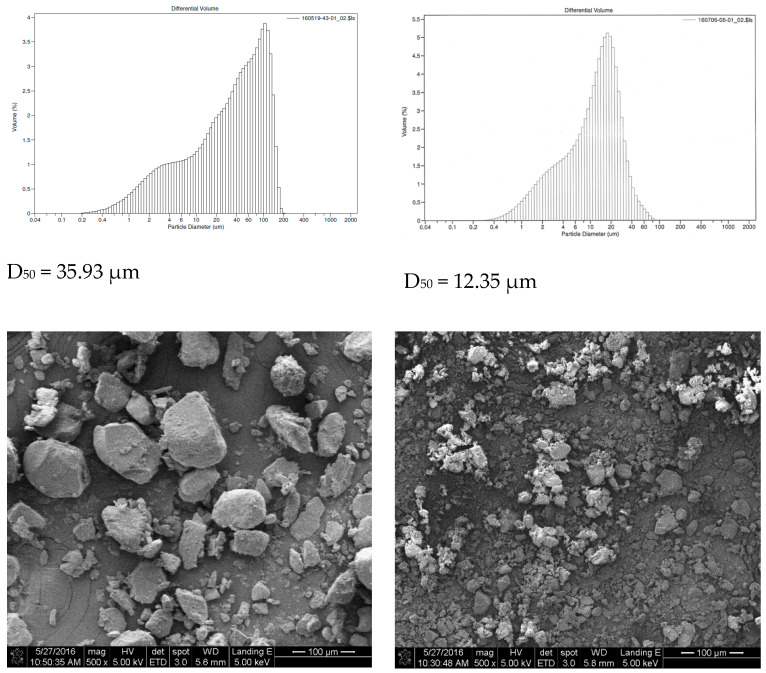
Particle size distribution of the HEM powder. D_50_ represents the mean volumetric particle size.

Our results and the corresponding electron microscope are shown in Figure 2. The volumetric mean diameters of the particles from two different batches were 35.93 μm and 12.35 μm, respectively.

### 2.3. HPLC Analysis

**Figure 3:**
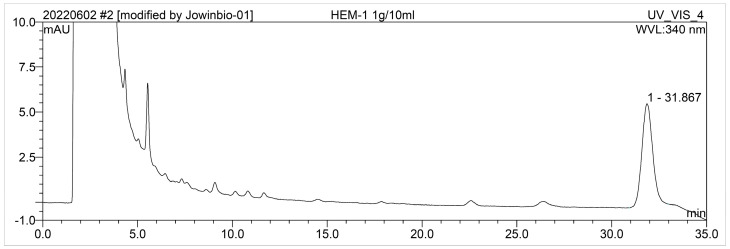
HPLC chromatogram of HEM. The retention time of the diterpenoid erinacine A peak was within the range of 31.000–33.000 min.

The chromatograms of HEM generated using HPLC are displayed in Figure 3. The retention time of 31.867 min corresponded to erinacine A, which was identified by comparison with prepared standards (kindly provided by Jowin Biopharma Inc., New Taipei City, Taiwan). The peak contents were quantified from the established calibration curve as erinacine A is 30 μg/g dry weight of HEM.

### 2.4. Neuroprotective Effects of HEM on MPTP-Treated Mice

**Figure 4:**
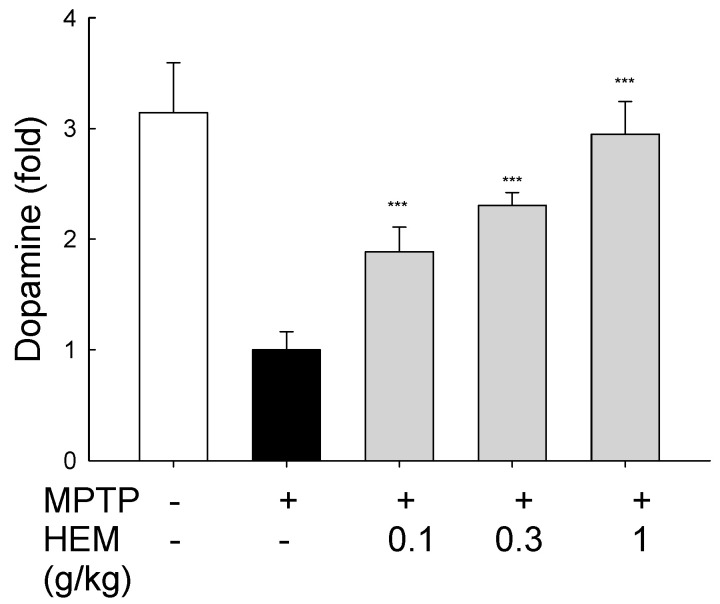
Dopamine levels in the substantia nigra of treated and untreated mice. A significant increase in the dopamine level was found in the HEM cotreated mice compared with the MPTP group in a dose-dependent manner. *** *p* < 0.001 compared with the MPTP-treated group.

To evaluate the neuroprotective effect of HEM on ameliorating MPTP-induced cytotoxicity and oxidative stress, the dopamine levels in the substantia nigra were determined. MPTP was the agent that decreased the dopamine level in the brain of mice to 1535 ng/g, as shown in Figure 4. Once coadministering mice with HEM powder at different levels, the dopamine level was increased to 2897, 3535, and 4527 ng/g at 0.1, 0.3, and 1.0 g/kg, respectively. The HEM powder could effectively increase the dopamine level in the brain to 2–3 times at 0.3 and 1.0 g/kg. Our findings indicate that the HEM powder was able to reverse the MPTP-induced dopamine reduction in the tested mice brain in a dose-dependent manner, as shown in Figure 4.

**Figure 5:**
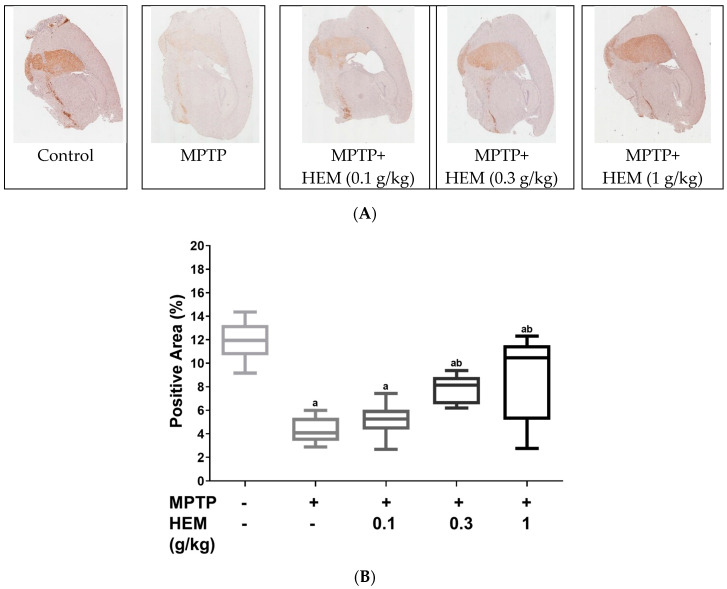
(**A**) Immunohistochemical staining of tyrosine hydroxylase (TH). The MPTP group exhibited dopamine deficiency syndrome. Co-treatment of HEM ameliorated MPTP-reduced TH expression in a dose-dependent manner. (**B**) The positive area of striatum is expressed in a Box and Whisker Plot. The results reflect the mean values of cells. The letter “a” represents a statistical difference (*p* < 0.01) compared to the control group, while the letter “b” represents a statistical difference (*p* < 0.01) compared to the MPTP treatment group. The statistical analysis was performed using Holm-Sidak tests.

We thereafter performed immunostaining for tyrosine hydroxylase (TH), the enzyme that catalyzes the rate-limiting step in the biosynthesis of dopamine, in the right cerebrum, and the results are shown in Figure 5. It was found that MPTP treatment could destroy neurons and the median percentage of the positive area decreased from 12% to 4%, as shown in Figure 5. However, HEM administration could restore MPTP-reduced TH-positive cells, and the median percentage of the positive area increased from 4% to 10% as the administration of HEM increased from 0.1 to 1.0 g/kg. These findings demonstrated that HEM possessed the ability to reverse MPTP-caused neurodegeneration.

### 2.5. Antioxidant Activity of HEM on the Brain

**Figure 6:**
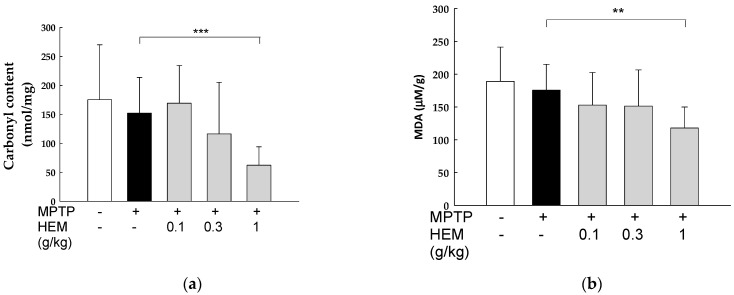
Effects of MPTP treatment in the presence or absence of HEM in the brains of mice on the (**a**) protein carbonyl content and (**b**) MDA levels. A significant decrease in the PC and MDA levels was found in the brains of mice obtained from treatment with a high concentration of HEM (1 g/kg) compared with the MPTP group. Values are expressed as mean ± SD. ** *p* < 0.01;*** *p* < 0.001 compared with the MPTP-treated group.

To further evaluate the antioxidant activities of HEM on the brains of MPTP-treated mice, oxidative stress biomarkers, including protein carbonyl (PC) content and malondialdehyde (MDA) levels in the homogenized brain, were evaluated. As shown in Figure 6, there was no difference in the PC levels in the brains of the MPTP-induced group compared with those in the control group. Similar results were also observed with respect to the MDA levels, as shown in Figure 6, which is the oxidative product of polyunsaturated fatty acids peroxidation. However, both PC and MDA levels decreased significantly in the MPTP + HEM (1 g/kg) group compared to the MPTP group (*p* < 0.01), as shown in Figure 6.

### 2.6. Antioxidant Activity of HEM on Livers

**Figure 7:**
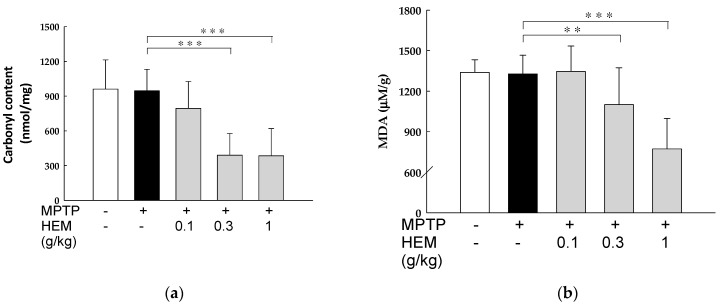
Effects of MPTP treatment in the presence or absence of HEM in the livers of mice on the (**a**) protein carbonyl content and (**b**) MDA levels. A significant decrease in the PC and MDA levels was found in the livers of mice treated with MPTP + HEM (0.3 and 1 g/kg) compared with the MPTP group. Values are expressed as mean ± SD. ** *p* < 0.01; *** *p* < 0.001 compared with the MPTP-treated group.

Our results showed that there was no difference in the PC and MDA levels of the MPTP-induced group compared with the control group. However, there was a significant reduction in both PC and MDA levels in the MPTP + HEM (0.3 and 1 g/kg) group compared to the MPTP group (*p* < 0.01 and *p* < 0.001), as shown in Figure 7.

### 2.7. Effect of HEM Treatment on Oxidative Stress Parameters of RBCs

**Figure 8:**
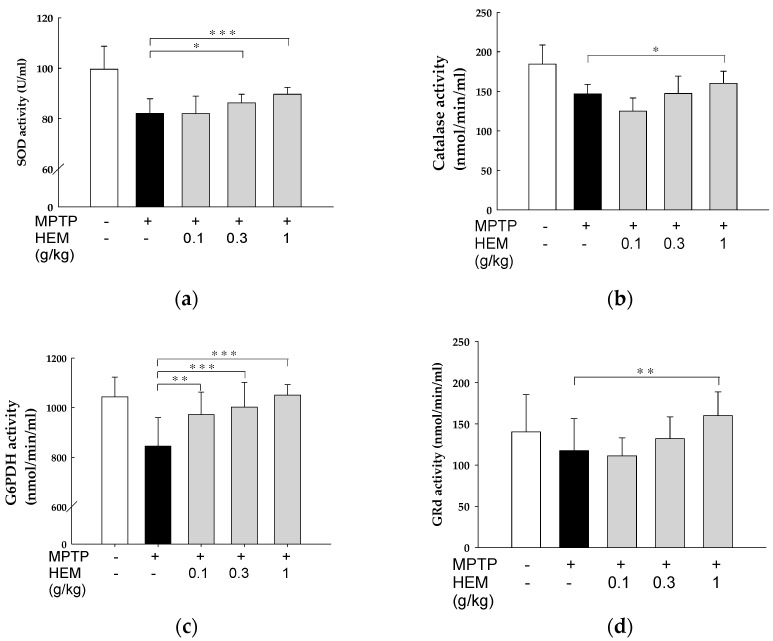
Effect of HEM treatment on the oxidative stress parameters in RBCs. Different parameters were determined, including (**a**) SOD, (**b**) catalase, (**c**) G6PDH, and (**d**) GRd. Values are expressed as mean ± SD. * *p* < 0.05; ** *p* < 0.01 and *** *p* < 0.001 as compared with the MPTP-treated group.

Oxidative stress biomarkers, such as SOD, catalase, G6PDH, and GRd, were evaluated in the red blood cells (RBCs) of male mice exposed to MPTP in the presence or absence of HEM at different concentration. As shown in Figure 8, the antioxidant biomarkers of RBC were reduced after MPTP treatment, although most differences were only slightly significant. However, HEM administration at different concentrations for 30 days could reverse the reduced MPTP-causing enzyme activities in a dose-dependent manner (Figure 8).

## 3. Discussion

**Figure 9:**
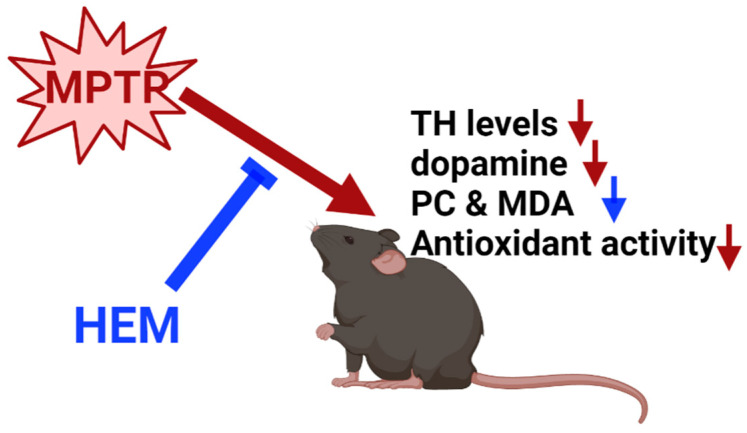
A schematic diagram of HEM preventing MPTP toxicity. Deep red represents the results of MPTP treatment; blue represents the results of HEM treatment. MDA, malondialdehyde; PC, protein carbonyl; TH, tyrosine hydroxylase. Deep red arrows represent the results of MPTP treatment; blue arrows represent the results of HEM treatment.

Herein, we established a micronized HEM powder using a spiral jet mill to break the cell walls and accelerate the release of active ingredients from HEM. Erinacine A (EA), the main natural antioxidant compound of HE, was 30 μg/g dry weight in HEM. HEM could increase the dopamine level in the brain, suggesting that HEM could recover the function of the substantia nigra. TH expression in the striatum was also recovered to almost full levels using HEM powder in MPTP-treated mice. This effect is, at least in part, due to reduced oxidative stress in the body (Figure 9).

Lipophilic MPTP can easily penetrate the blood–brain barrier (BBB) and is then converted into 1-methyl-4-phenylpyridinium (MPP+) by enzyme monoamine oxidase B, which activates cell death signaling pathways and induces dopaminergic neurotoxicity [23]. The present study demonstrated that MPTP administration (20 mg/kg/day for 5 days) reduced dopamine release and TH expression in the striatum, indicating successful induction of dopaminergic neurotoxicity in mice. Furthermore, video evidence (Supplementary Materials Video S1) confirmed the success of the behavioral model. These results are consistent with previous experiments [24] and confirm the validity and efficiency of our animal model. However, it should be noted that protein oxidation levels did not significantly change after administering 20 mg/kg of MPTP. Possible reasons for this include: (1) oxidative stress is an early indicator of cell damage, and the cellular damage caused by MPTP may gradually diminish over time. In our study, the observation period was longer (25 days) compared to the usual period (20 days). Additionally, (2) the dosage of MPTP we administered was lower, and (3) younger mice tend to have better recovery characteristics [25,26]. In our current study, the oral administration of HEM could restore MPTP-induced dopaminergic neuron degeneration and reduced dopamine levels. It is common knowledge that PD is caused by the loss of nerve cells in the patient’s brain, leading to the reduction of dopamine levels, which plays a vital role in regulating body movement. Therefore, the present findings may suggest that HEM could be beneficial to increasing the dopamine levels in patients with PD. Shimbo et al. have found that erinacine A, a bioactive compound in HEM, stimulates the secretion of the nerve growth factor, an essential protein for supporting neuron’s growth and maintenance, in the rat locus coeruleus. Moreover, erinacine A has also been shown to stimulate dopamine metabolites production [27]. These experimental results are also consistent with our experiments.

MPTP neurotoxicity is rapid (as early as 2 h) and stabilizes within 7 days. In addition, 90% of striatal dopamine depletion and 70% loss of dopaminergic neurons were induced after four injections of MPTP at a daily dose of 20 mg/kg, thereby causing motor deficits [28]. MPTP neurotoxicity is associated with the inhibition of ATP production and stimulation of multiple ROS production, which then declines and damages protein function through oxidation and nitration [24]. In SY5Y neuroblastoma cells, MPTP (50 μM) exposure stimulates intracellular ROS production, reaching its peak at 6–12 h, and then declining to near baseline after 48 h of exposure [29]. ROS are known to play a key role in the aging process and have also been implicated in aging-related neurodegenerative diseases such as PD [29]. Therefore, reinforcement of the antioxidant defense system or scavenger administration is critical because it may combat these diseases [30]. Furthermore, reducing free radicals via antioxidants has been shown to combat toxin-induced degenerative diseases [20]. Herein, we found that HEM significantly reduced free radical production both in the brain and liver. In addition, the antioxidant activity was significantly increased with the oral administration of HEM powder.

The brain is easily affected by the aging processes caused by oxidative stress. Our experimental study showed that MDA production was reduced in the HEM (0.1 and 0.3 g/kg) groups compared with the control and MPTP-treated groups, although this effect was not significant at low doses of HEM. The administration of high doses of HEM (1 g/kg) could significantly (*p* < 0.01) reduce MDA levels. Similar results were also found regarding the increase in PC contents, suggesting that HEM could effectively counteract oxidative stress in brain tissues. Furthermore, hepatic MDA and PC levels were also reduced in MPTP-treated mice. Antioxidative stress parameters, including SOD, catalase, G6PDH, and GRd activities, were elevated in RBCs of the MPTP + HEM treatment group compared with the MPTP-treated group.

HE possesses neuroprotective effects and its bioavailability can be determined using erinacine A and erinacine S, its two major compounds. Erinacine A can be detected in plasma at 1 min after the oral administration of HE as it penetrates the BBB via passive diffusion. Consequently, it was detected in the brain 4 h post-administration and reached its maximum level after 8 h. Moreover, the binding of erinacine A was found to be the highest (28.94% ± 9.29%) in the brain. The absolute bioavailabilities of erinacine A and erinacine S were 24.39% and 15.13%, respectively [31,32].

Conclusively, HEM powder can be very beneficial in combating diseases that follow dopaminergic pathways in the brain, including nigrostriatal, mesolimbic, mesocortical, and tuberinfundibular systems that play vital roles in regulating many important physiological functions. This study also found that HEM could reduce ROS levels in the brain, liver, and blood. ROS are mainly produced by the mitochondria during both physiological and pathological conditions, and by endothelial and inflammatory cells. Despite the fact that these organelles have intrinsic ROS scavenging capacities, these may not be enough to address the cellular need of clearing ROS generated by the mitochondria [33]. Hence, HEM powder enrichment may provide answers to this question, and thus protect individuals’ wellness and health from ROS-induced cellular damages.

## 4. Conclusions

In conclusion, the study found that HEM powder has the potential to fight diseases that affect the dopaminergic pathways and lower ROS levels in the brain, liver, and blood, thus safeguarding individuals from cellular damage caused by ROS. HEM enrichment may address the cellular need for clearing ROS generated by the mitochondria, thus protecting individuals’ health and wellness from ROS-induced cellular damage.

## 5. Materials and Methods

### 5.1. Preparation of HE Mycelium

HEM powder was purchased from Fungus Biotech, Co., Ltd., Yilan, Taiwan, which used the HE strain (BCRC 36470, Bioresource Collection and Research Center, Hsinchu, Taiwan) and was produced under solid-state fermentation. The HEM powder was dried at 60 °C in a tray dryer and grinded into 100-mesh powder at Fungus Biotech. It was then further ground into smaller particles through a spiral jet mill (spiral jet Mill, OM2 Micronizer, Sturtevant, Int., Hanover, MA, USA) to undergo the cell wall-breaking effect with a particle size distribution of D75 < 50 μm micronized powder at Formosan Nano Biology Co., Ltd., Taichung, Taiwan. The cell wall-breaking technology greatly contributes to the increased rate of releasing active ingredients from the fine HEM powder.

### 5.2. Particle Size Analysis

HEM particle size distributions were evaluated using a Beckman Coulter LS230 particle size analyzer, which can measure particles ranging from 40 nm to 2 mm in size [34]. HEM particles were measured in an ethanol dispersed solution.

### 5.3. High Performance Liquid Chromatography (HPLC)

We weighed 1 g of the *Hericium erinaceus* mycelium powder and extracted it with 5 mL of 50% methanol using ultrasonic technology. The resulting mixture was then centrifuged at 3000× *g* for 5 min, and this procedure was repeated once. The supernatant was filtered using ADVANTEC NO.1 membranes and diluted with 50% methanol to a final volume of 10 mL. Prior to HPLC analysis, the solution was filtered through a 0.22 µm PVDF syringe filter and degassed.

HPLC analysis of erinacine A was performed on a Thermo Scientific Dionex Ultimate 3000 HPLC system (Thermo Scientific, Bremen, Germany) equipped with a quaternary rapid separation pump (LPG-3400SD), TCC-3000 temperature-controlled column (40 °C), and DAD-3000 diode array detector, as previously described, with minor modifications [35]. Chromatographic separations were achieved on an InertSustain C-18 (250 × 4.6 mm, 5 μm) with a linear A–B gradient (0–20 min 66% B to 70% B, 25–35 min 70% B to 100% B) at a constant flow rate of 1 mL/min and a total run time of 35 min. Solvent A consisted of 0.2% H_3_PO_4_ in Milli-Q water and solvent B of 100% methanol. The absorption spectra of eluted compounds were detected at 340 nm using Dionix Chromeleon software (Version 6.80, Service Release SR14).

### 5.4. Animals Groups and Experimental Procedure

Adult (8–12 weeks old) male C57BL/6Narl mice, weighing 20–30 g, were purchased from the National Laboratory Animal Center (Taipei, Taiwan). The animals were housed at a temperature of 22 ± 1 °C, with 14 h of automatic illumination daily (06:00–20:00) in the Animal Center of the National Yang-Ming University, Taiwan. Animal care conformed to the Guidelines of the Animal Use and Care Committee of National Yang-Ming University, Taiwan. Food and water were available ad libitum. The animal use protocol was approved by the Institutional Animal Care and Used Committee (Approval Number: 1050306 & 1060509).

The MPTP model of PD was induced, as described previously [22]. Mice were randomly assigned into five groups, as shown in Figure 1: the control group, the MPTP group (20 mg/kg/day for the first 5 days; Tokyo Chemical Industry, TCI), and MPTP + different dosages of HEM groups (0.1 g/kg, 0.3 g/kg, and 1 g/kg, respectively). Mice received an intraperitoneal (i.p.) injection of MPTP, and the same quantity of saline was given in the control group. Mice were orally gavaged with H_2_O or HEM for 30 days.

### 5.5. Dopamine Measurement

Mice were sacrificed, and the striatum was quickly dissected on ice and homogenized in a stock solution containing 0.1 M HClO_4_, 0.1 mM EDTA, and 0.1 mM Na_2_S_2_O_5_ and centrifuged at 13,000 rpm for 10 min at 4 °C. The supernatant was filtered with 0.45-μm membranes before HPLC analysis. The dopamine level in this isolated substantia nigra homogenate was measured with electrochemical detection, as described previously [22].

### 5.6. Tyrosine Hydroxylase Measurement

Tyrosine hydroxylase (TH), a key precursor for dopamine production, was measured using immunohistochemistry (IHC) [36,37]. The right cerebrum was immersed in cold paraformaldehyde in 0.1 M phosphate buffer (pH: 7.4) and sectioned into 10 μm thick slides. All sections were stained for TH determination. The optical density of areas with TH expression was determined by measuring at least three randomly selected microscopic fields on each slide. The average integral optical density was defined as the percentage of positive area × optical density/total area [38].

### 5.7. Protein Carbonyl Content Measurement

The liver and brain of the tested mice were collected, and the protein carbonyl content was then evaluated [24]. Approximately 150–200 mg of brain or liver tissues were homogenized separately in 50 mM of MES buffer (1–2 mL, pH 6.7, containing 1 mM EDTA) and centrifuged. Each supernatant was collected and stored at −80 °C. The sample was then determined using a protein carbonyl colorimetric assay kit (No. 10005020, Cayman, MI, USA).

### 5.8. Lipid Peroxidation Level Determination

The lipid peroxidation level in the brain and liver was determined as described in a previously published method [39] and expressed as the MDA value. MDA was measured with a thiobarbituric acid-reactive substance (TBARS) assay kit (Item No. 10009005, Cayman, MI, USA). Briefly, the brain and liver were isolated and homogenized in cooled RIPA buffer. Consequently, all samples were centrifuged at 1600× *g* for 10 min at 4 °C. The supernatant was then stored at −80 °C, and the MDA values were determined.

### 5.9. Antioxidant Status Activity

Antioxidant enzyme activities of RBCs were evaluated using a previously published method [40]. Whole blood with heparin was collected and centrifuged. RBCs were washed with normal saline twice and lysed using 50 mM phosphate buffer (pH: 6.6). The supernatant was collected and determined within one month. Superoxide dismutase (SOD, Item No. 706002), catalase (CAT, Item No. 707002), glutathione peroxidase (GPx, Item No. 703102), glutathione reductase (GRd, Item No. 703202), and glucose-6-phosphate dehydrogenase (G6PDH, Item No. 700300) activities were determined using commercial kits (Cayman, MI, USA).

### 5.10. Data Analysis and Statistical Assessment

Data collected were expressed as mean ± SD. Analysis of variance was used to access the statistical significance for repeated data measurements, and the differences among individual mean values in different groups were analyzed using the Holm-Sidak post-hoc test followed by one way Analysis of Variance (ANOVA). Differences were considered to be significant at *p* < 0.05.

## Data Availability

The data presented in this study are available on request from the corresponding author.

## Supplementary Materials

The following supporting information can be downloaded at: https://www.mdpi.com/article/10.3390/molecules28083386/s1, Video S1.

## Sample Availability

Samples of the compounds will not be available.
